# Proceedings of the Hahnemannian Association of Pennsylvania

**Published:** 1888-03

**Authors:** Wm. Jefferson Guernsey

**Affiliations:** Secretary


### PROCEEDINGS OF THE HAHNEMANNIAN ASSOCIATION OF PENNSYLVANIA.

The regular monthly meeting of the Hahnemannian Association of Pennsylvania was held Tuesday, February the 14th. In the absence of the President, Dr. Preston, the Vice-President, Dr. C. Carleton Smith, occupied the chair.

Dr. Smith read paragraph 275 from the *Organon* and made some remarks upon its tenor, the chief topic of discussion being as to whether or not Hahnemann advised us against giving a large dose of a potentized drug. Dr. Smith thought Hahnemann did warn us against possible aggravations from such overdosing. Hahnemann frequently speaks of giving one or two pellets of a remedy.

Dr. Lee—When Hahnemann speaks of the size of the dose, does he refer to the potency or to the amount of the drug?

Dr. Smith—I understand him here to refer to the size of the dose.

Dr. G. H. Clark—I think he means to tell us that we may give too much of a potentized drug at a dose.

Dr. Lee—Suppose the drug is put into half a glass of water; does it make any difference whether one or two spoonfuls are taken at a dose?

Dr. Smith—As the larger amount of water would cover a larger surface, I believe there would be greater medicinal action, hence more probability of an aggravation.

Dr. R. B. Johnstone—I had recently a letter from Dr. A. S. Kimball, telling me of a case in which Dr. Wm. P. Wesselhœft had given Spongia$^{cm}$. The patient grew worse, so the doctor gave Spongia$^{cmm}$. The patient continuing to get worse, he gave the remedy dissolved in water with prompt improvement. This case does not indorse Dr. Smith's views.

Dr. Smith—As a general rule, more prompt and vigorous action will follow the administration of the remedy in water.

Dr. G. H. Clark, in speaking of treating patients suffering from *acute* or recent mercurial poisoning, referred to the fact that the Mercury was held in the bones, and was therefore constantly being re-absorbed, producing a constant action on the patient for a long time after he had ceased to take the drug. To counteract this the dose of the antidote would generally have to be strong. In treating cases of *chronic* mercurial poisoning, the higher potencies would be the best.

Dr. J. V. Allen asked if cases of acute mercurial poisoning could not also be cured by potencies. He thought they could and would be willing to risk it.

Dr. Johnstone—I once consulted Dr. Biegler, of Rochester, N. Y., in regard to my health. He examined my mouth and advised the removal of all the amalgam fillings, after which there was no difficulty in effecting a cure of my troubles.

Dr. J. V. Allen—Can any one tell me if in his experience the rapid eating away of tissues by a phagedenic chancre can be stopped by the use of the potentized remedy? I know of a physician who would join this Association but for the fact that he fears to give up the use of local applications on these chancres.

Dr. G. H. Clark—I once saw a man having lupus, which was eating away the tissues very rapidly. Lyc. stopped the destruction promptly, leaving a clearly marked line to show where it had extended.

Dr. R. B. Johnstone—In New York State I once had medical charge of a gang of Italian workmen. There I saw a great number of phagedenic chancres and used no other treatment than the indicated remedy in a high potency. Most of the cases called for Corallium rub., on account of the excessive tenderness and bleeding from the least touch. Corallium in the 50 M or CM would stop the destructive process in about twelve hours.

Dr. Horace Still, of Norristown, who had been appointed to prepare a paper on a drug, was not able to be present, but sent a paper on Ambra grisea, which was read. (See another page for the article.)

Dr. Lee—The mention of falling off of the hair reminds me that Wiesbaden water is a remedy for this trouble. Under its use the hair falls out and is followed by a profuse and rapid growth of new hair. Another great remedy is the onion (Allium cepa.). Old Hippocrates advised one to rub his bald spots with a raw onion. I would also refer to another symptom of Ambra, its profuse urination; the patient passes much more water than he drinks. Aurum and Mercury have this profuse urination also. It will be found so stated in Dr. Lippe's *Materia Medica.*

Dr. R. B. Johnstone wanted to know what remedies had the sensation as though the object observed were moving before the eyes.

Dr. G. H. Clark—Physostigma.

Dr. Lee—The range of vision changing while reading is Agar. and Jabor. The type seeming to move, is Agar., Amm. c., Cic., Con., Merc., Phys.

Dr. Clark offered a resolution that the members of the I. H.

A. be hereafter admitted as *active* members on receiving a unanimous vote. Also that all motions to amend the Constitution be required to lay over one month only, instead of two, as now the rule. Laid over until April meeting.

Appointees for March meeting:

*Materia Medica.*—Dr. Edmund J. Lee; subject, Characteristics of Ten Tissue Remedies.

*Organon.*—Dr. Walter M. James.

*Original paper.*—Dr. J. W. Thatcher.

A letter of regret at his unavoidable absence was received from Dr. Lawson. The preamble and resolutions relative to the death of Dr. Adolph Lippe (which had been adopted at a special meeting and published in the Philadelphia *Ledger* and THE HOMŒOPATHIC PHYSICIAN) were reported. Adjourned.

WM. JEFFERSON GUERNSEY, *Secretary.*

